# In females with anterior knee pain, the infratuberositary contribution to external tibial torsion increases with torsion severity and does not correlate with tibial tubercle lateralisation

**DOI:** 10.1002/jeo2.70603

**Published:** 2025-12-28

**Authors:** Vicente Sanchis‐Alfonso, Cristina Ramírez‐Fuentes, Jose Yañez‐Rodríguez, Laura Parra‐Calabuig, Marcos López‐Vega, Julio Domenech‐Fernandez

**Affiliations:** ^1^ Department of Orthopaedic Surgery Hospital Arnau de Vilanova Valencia Spain; ^2^ Department of Radiology Hospital Universitario y Politécnico La Fe Valencia Spain

**Keywords:** anterior knee pain, patellofemoral pain, pathological external tibial torsion, rotational tibial osteotomy, tibial tubercle, torsional abnormalities

## Abstract

**Purpose:**

To perform a segmental analysis of tibial torsion in females with refractory anterior knee pain (AKP) and investigate the relationship between external tibial torsion (ETT) and the position of the tibial tubercle (TT).

**Methods:**

All disabling AKP patient refractory to adequate physical therapy, that presented to our clinic between January 2013 and December 2024 were retrospectively reviewed. Inclusion criteria: (1) females, (2) ≥18 years old, (3) torsional CT scan performed for strictly clinical reasons. Patients were classified into three groups according to ETT: normal (≤30°), moderate (31°–40°), and severe (>40°). ETT was measured proximally and distally to the TT, and the contribution of distal tibial torsion (DTT) to total tibial torsion (TTT) was calculated. TT lateralisation (TTL) relative to the tibial plateau′s maximum transverse diameter was measured. Statistical analysis for comparisons was conducted using one‐way ANOVA with Bonferroni correction and Student′s *t*‐test. Correlations were assessed with Pearson′s coefficient. To evaluate the association between TTT and the percentage of DTT, a linear regression analysis was performed. Inter‐observer reproducibility was evaluated using the intraclass correlation coefficient. Statistical significance was set at *p* < 0.05.

**Results:**

A total of 197 tibial CT scans from 101 AKP females were analysed. The percentage of contribution of DTT to TTT was significantly higher in the severe group (35.2%, SD 11.5) compared to the moderate (22.1%, SD 12.1) and normal (18.2%, SD 16.7) groups (*p* < 0.01). A moderate correlation between TTT and DTT contribution was identified (*R* = 0.540, *p* < 0.001). Linear regression analysis indicated that ETT was a significant predictor of DTT contribution (*p* < 0.001). No significant correlation was found between TTL and ETT severity.

**Conclusion:**

The infratuberositary contribution to pathological ETT increases with the severity of the torsion. Moreover, the degree of ETT does not affect TTL.

**Level of Evidence:**

Level IV.

**Abbreviations**
AKP: anterior knee pain
CT: computed tomography
DTT: distal tibial torsion
ETT: external tibial torsion
MRI: magnetic resonance imaging
PACS: picture archiving and communication system
PTT: Proximal tibial torsion
TT: tibial tubercle
TTL: tibial tubercle lateralisation
TTT: total tibial torsion
TT–PCL: tibial tubercle–posterior cruciate ligament
TT–TG: tibial tuberosity–trochlea groove

## INTRODUCTION

Pathological external tibial torsion (ETT) is recognised as a contributing factor to anterior knee pain (AKP), with favourable outcomes reported following rotational tibial osteotomy [4, 6, 7, 8, 9, 12, 16, 24, 25, 26, 30, 33, 37, 38]. However, despite good outcomes after rotational tibial osteotomy, this surgery is not widely used and has very controversial aspects. One of the most controversial aspects of this surgery is determining the optimal level for performing the osteotomy. Most authors defend that the location of osteotomy should be supratuberositary because it can correct the Q‐angle or the TT‐TG distance when it is excessive [6, 8, 9, 12, 16, 24, 25, 26, 30, 33] and only some maintain that it should be infratuberositary [4, 14, 37, 38]. Moreover, supramalleolar osteotomy is an increasingly used alternative for ETT correction, avoiding proximal muscle detachment and potential peroneal nerve injury [40]. Moreover, another reason for a supramalleolar osteotomy is to avoid growth plates and therefore is often used in children.

This is an investigational anatomic study whose main objective is to address the factors influencing the choice of osteotomy level in young AKP female patients with pathological ETT. Theoretically, an osteotomy should be performed at the level most contributing to the deformity, making it essential to define the origin of pathological ETT. Therefore, the main objective of this imaging‐based study is to perform a segmental analysis of tibial torsion in AKP patients, comparing those with normal and pathological ETT. The medical literature is inconclusive regarding the origin of the deformity. In 2020, Winkler et. concluded that ETT was an infratuberositary deformity (tibial segment inferior to the tibial tuberosity) [42]. However, in 2022, Yi Qiao et al. concluded that both the proximal and distal segments were major contributors to excessive ETT [31]. Our working hypothesis was that pathological ETT is an infratuberositary deformity. However, the critical factor in determining the osteotomy level is not solely the origin of the deformity but whether the tibial tubercle (TT) is lateralized in patients with pathological ETT compared to a control group. If the TT is lateralized, it would make sense to perform a supratuberositary osteotomy. Conversely, if the TT is not lateralized, performing a supratuberositary osteotomy would result in excessive medialization of the TT, potentially causing medial femorotibial and patellofemoral joint overload, which could lead to osteoarthritis in the long term [23, 27]. Our second working hypothesis was that TT is not lateralized in the vast majority of AKP patients.

## MATERIAL AND METHODS

### Participants and study design

This study was approved by our institutional review board (CEIm Hospital Arnau de Vilanova, Valencia, Spain; Protocol #PI 21_2024). From January 2013 to December 2024, 203 consecutive patients who had been diagnosed with AKP resistant to an adequate conservative treatment performed by a group of 4 physical therapists specialising in treating patellofemoral pain were eligible for this study. For AKP patients with disabling pain and severe disability not responding to conservative treatment, weight‐bearing whole‐limb anteroposterior view radiograph, computed tomography (CT) for evaluation of torsional abnormalities and magnetic resonance imaging (MRI) were performed in all of them as part of a routine radiographic workup. In our cohort females predominated and therefore we include females only (180 patients) to reduce potential bias in the results. Torsional CT studies performed in this cohort of patients were retrospectively collected to evaluate the torsional morphology of the tibia and its association with the position of the TT. All CT studies were performed and assessed by the same musculoskeletal radiologist (C.R‐F). In all cases, the torsional CT study was performed for strictly clinical reasons following the diagnostic protocol of the Knee Unit of our Department.

Inclusion criteria: (1) females, (2) ≥18 years of age, (3) AKP refractory to an adequate physical therapy treatment in which a torsional CT scan of the lower limbs was done to complete the diagnosis and plan the definitive treatment and (4) patients with CT images that include sections of the entire TT. All patients included in the study had imaging studies (radiography and MRI) that excluded associated lesions (osteoarthritis, chondropathy grade III and IV according to ICRS – International Cartilage Repair Society ‐ classification, meniscal or ligamentous injury, patellar instability and trochlear dysplasia grade B, C and D according to Dejour′s classification). Exclusion criteria for the present study were: (1) male, (2) <18 year of age, (3) previous surgery in the knee or tibia, and (4) history of tibia fracture. Five tibiae were excluded because CT image that did not include the entire TT. Finally, 101 patients (197 tibiae) were eligible for this study.

According to the literature, the average ETT in women is 27° [43]. Most authors use 30° of ETT associated with clinical symptoms as the cut‐off point to perform a rotational tibial osteotomy [8, 12, 15, 20, 21]. On the other hand, according to other authors, osteotomy is indicated when the ETT is greater than 40° [22]. A torsional deformity greater than 40° in combination with disabling AKP and severe disability is a relevant indication for operative correction in the opinion of the first author (V.S‐A). Therefore, we have designed a cross‐sectional study in which three groups of patients are created based on the value of ETT: Group 1 = patients with normal ETT (≤30°), Group 2 = patients with moderate ETT (between 31° and 40°) and Group 3 = patients with severe ETT (greater than 40°). Because of ethical issues of radiation exposure, the CT scans of normal ETT group was collected from those AKP patients in which for medical reasons a rotational study was needed as part of her medical assessment and the ETT in any of the two extremities resulted to be ≤30°, but not from healthy subjects.

### CT protocol

CT images were acquired on a high–spatial resolution 256–detector row CT scanner (Brilliance iCT; Philips). The patients were placed in a supine position with their hip and knee joints fully extended and their feet in 15° of external rotation. Three scans were obtained on each patient of the hips (scan range from the upper edge of the femoral heads to immediately distal to the lesser trochanters), knees (upper edge of the patella to immediately distal to the TT) and ankles (tibial plafond and both malleoli). The raw data sets acquired were 64 × 0.625 mm collimation, 0.5 s rotation time, 0.9 mm slice reconstruction thickness, 0.45 pitch, 120 kV and automated mAs control.

Tibial measurements were performed manually using the imaging tools on the advantage workstation 4,5 software (GE HealthCare) integrated in the picture archiving and communication system (PACS). All images in the present study have been previously anonymized. Once anonymization was carried out, a first examiner, a musculoskeletal radiologist over 15 years in practice (C.R‐F) performed all measurements and included them in the database. Subsequently, and independently, a second examiner, a radiologist resident in his last year of training (J.Y‐R) performed the same measurements and included them in the database, thus allowing for inter‐observer correlation to be studied. ETT was measured both proximally and distally to the TT and the percentage of contribution of distal tibial torsion (DTT) to the total tibial torsion (TTT) was calculated. The value of external tibial torsion (ETT) and total tibial torsion (TTT) is the same. When we refer to it as a pathological entity, we classify it as ETT. When we quantify it, we refer to TTT.

### Tibial torsion measurement. segmental analysis of the tibial torsion

**Figure 1:**
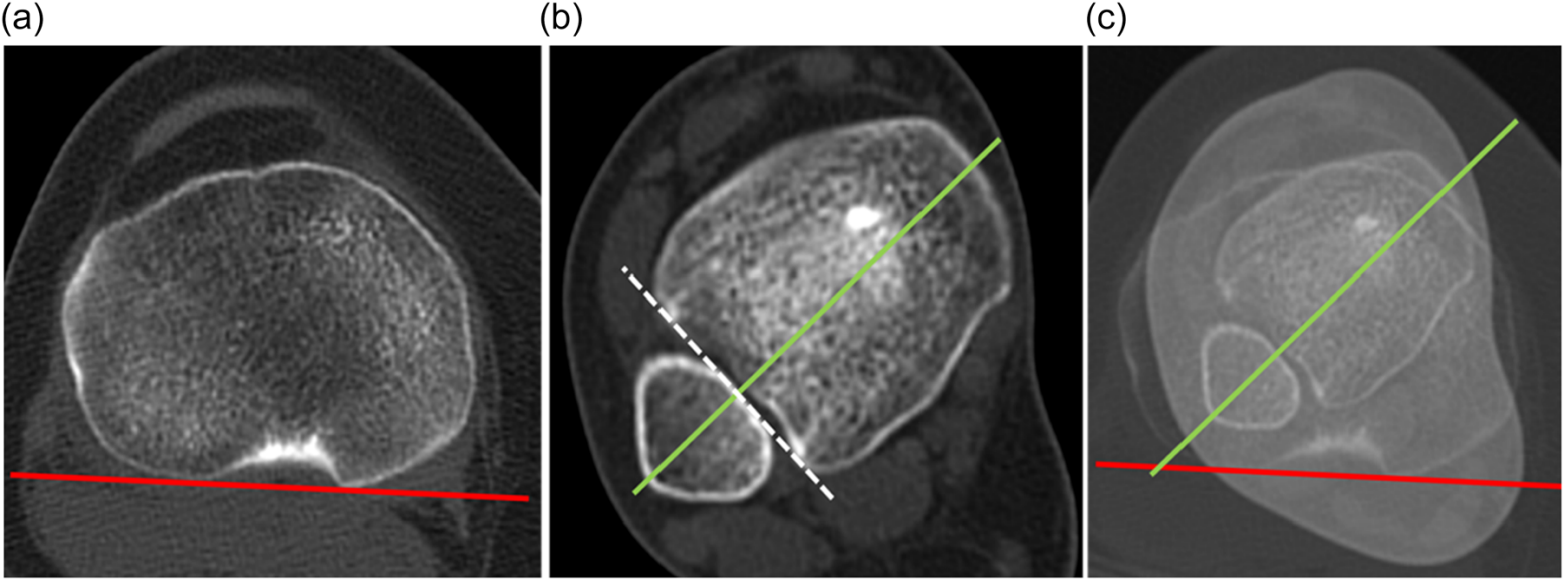
TTT measurement. (a) Axial CT image of the tibial plateau. The tangent to the posterior border of the tibial plateau forms the proximal line of reference (red line). (b) Axial CT image of the distal plafond and ankle malleoli. The line joining the most protruding part of the lateral and medial malleolus (green line), perpendicular to the fibular notch of the tibia (white dotted line) is the distal line of reference (green line). (c) Addition CT image of both tibial plateau and malleoli. Tibial torsion is the angle formed between the two lines. CT, computed tomography; TTT, total tibial torsion.

TTT was assessed using a technique modified from Jend et al. [20] The posterior condylar axis (tangent to the more proximal tibial plateau, where the posterior condylar notch was clearly recognised) constituted the proximal reference line (Figure 1). The distal reference line was formed by joining the most protruding part of the lateral and medial malleolus, perpendicular to the fibular notch of the tibia.

**Figure 2:**
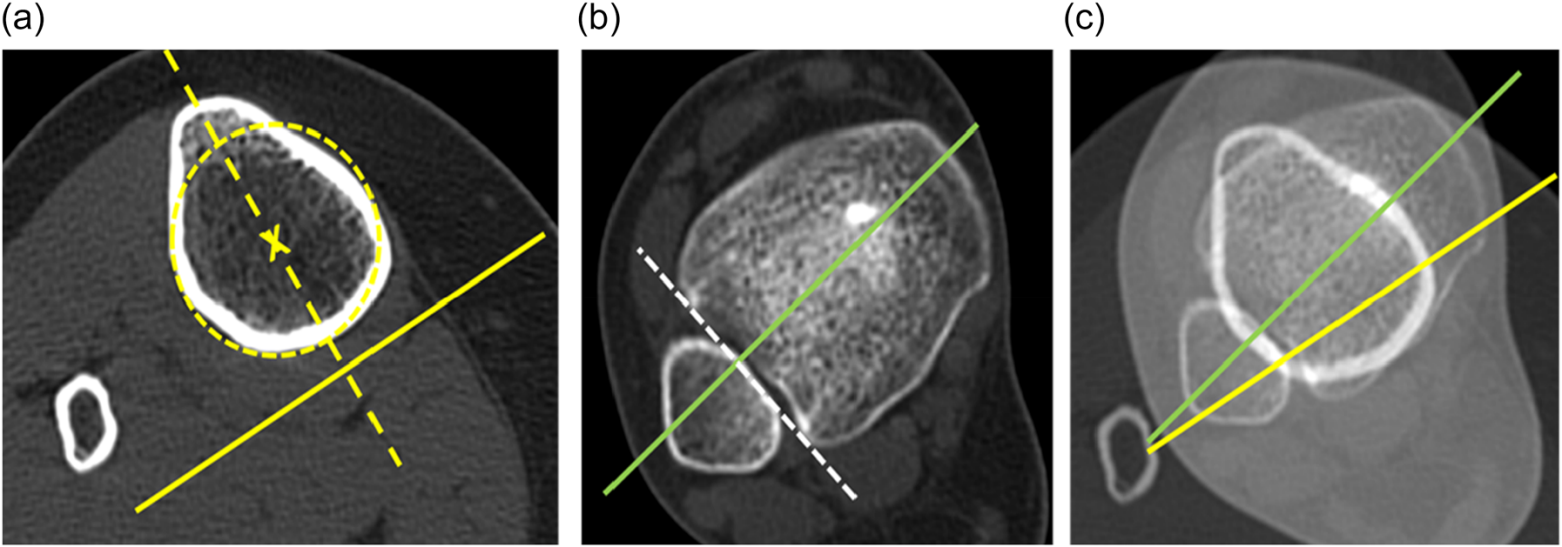
DTT measurement. (a) Axial CT image of the tibia, immediately distal to the TT. The line perpendicular to the line that connects the more anterior point of the tibia (dotted line) with the centre of the circle adapted to the posterior, medial and lateral borders of the tibia represent the proximal line (yellow line). (b) Axial CT image of the tibial plafond and malleoli. The bimalleolar axis is the distal reference line (green line). (c) Addition CT image of both infratuberositary tibia and ankle malleoli. DTT is the angle formed between the two lines. CT, computed tomography; DTT, distal tibial torsion; TT, tibial tubercle.

DTT was measured on the axial slice through the proximal tibial diaphysis, immediately distal to the TT where the insertion of the patellar tendon was not visible. This proximal reference line was taken as a line that is perpendicular to a line intersecting the more anterior point of the tibia with the centre of the circle adapted to the posterior, medial and lateral borders of the tibia (Figure 2). The distal reference line was the same as in TTT (bimalleolar axis). Proximal tibial torsion (PTT) was calculated as the subtraction between the TTT and the DTT.

### Position of the tibial tuberosity. Tibial tubercle lateralisation measurement

**Figure 3:**
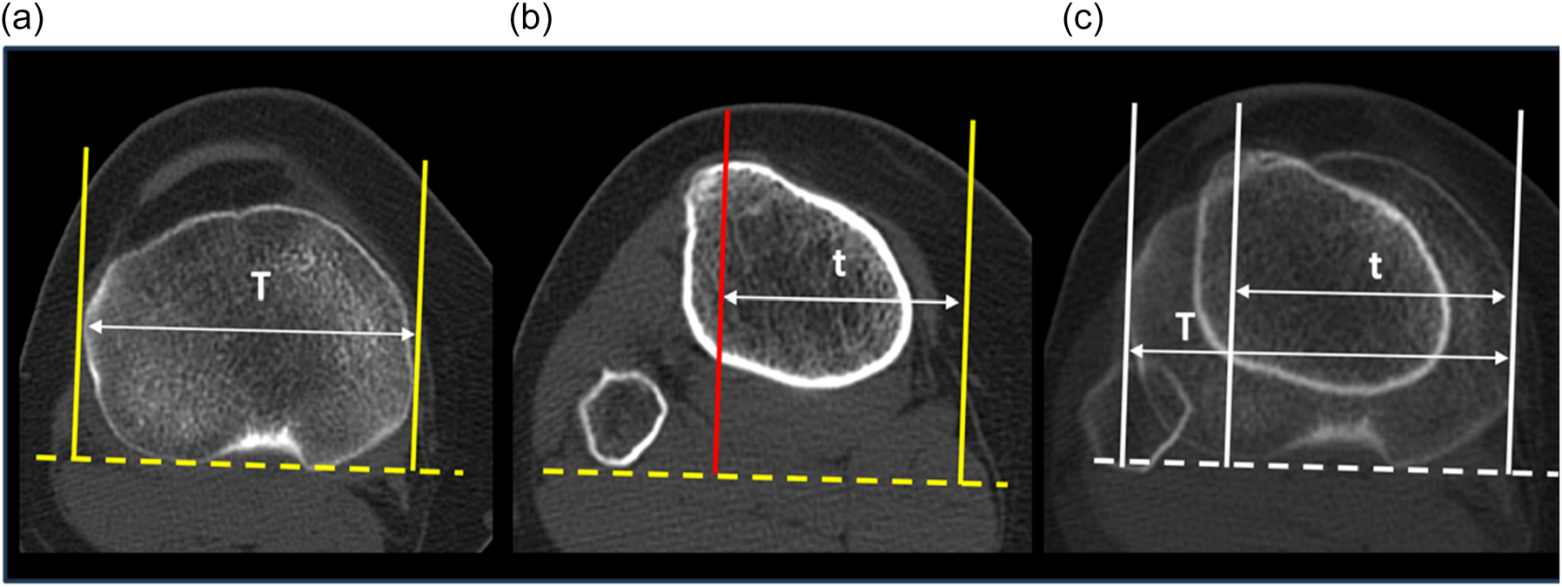
TT lateralisation measurement. (a) Axial CT image of the tibial plateau. The width of the tibial plateau (distance T) is the distance between the two lines perpendicular to the posterior bicondylar line passing through the medial and lateral border of the tibial plateau. (b) Axial CT image of the TT. The distance between the centre of the TT and the medial border of the proximal tibial condyle (distance t) was measured. TT lateralisation was calculated as t ÷ T. (c). Addition CT image. CT, computed tomography; TT, tibial tubercle.

TT lateralisation relative to the maximum transverse diameter of the tibial plateau and expressed as a ratio of this transverse diameter was assessed using the method of Ando et al. [1] modified by Tensho et al. [39]. The width of the proximal tibial condyle was measured in the slice that included the more proximal tibial plateau, where the posterior condylar notch was clearly recognised, as the distance between the two lines perpendicular to the posterior bicondylar line passing through the medial and lateral border of the tibial plateau (distance T). Then the distance between the midpoint of the TT and the medial border of the proximal tibial condyle (distance t) was measured. TT lateralisation was calculated as t ÷ T (Figure 3).

### Sample size calculation

A power analysis determined the number of required patients. Sample size was determined using G*Power (Version 3.1) for a one‐way analysis of variance (ANOVA). The study includes three groups based on tibial torsion (normal: ≤30°, moderate: 31°–40°, and severe: >40°) and aims to compare the percentage contribution of DTT to TTT among these groups. Preliminary data indicated that the mean distal contribution in normal individuals was 22%, with a standard deviation of 14%. A difference of half standard deviation was assumed as clinically relevant, following standard practice when no prior data define meaningful differences. To detect a difference of 0.5 SD in proportions, we used the following parameters: an effect size (Cohen's *f*) of 0.5, an alpha level of 0.05, and 80% power. Based on these assumptions, the required sample size was calculated to be 22 participants in the smallest group of the study.

### Statistical analysis

Statistical analyses were performed to evaluate differences and associations among groups. Comparisons between the three groups were conducted using a one‐way ANOVA with Bonferroni post hoc correction for multiple comparisons. The presence of normality in the data was tested with the Kolmogorov‐Smirnov test. The difference in TTL values between the normal and pathological groups was assessed using Student′s t‐test. The relationship between the TTT and the percentage contribution of DTT was analysed using Pearson′s correlation coefficient. To evaluate the association between TTT values and the percentage of DTT, a linear regression analysis was performed with a least‐squares method for curve fitting. Inter‐observer reproducibility was assessed using the intraclass correlation coefficient (ICC) with two factors and total concordance. ICC values were interpreted as follows: >0.90 indicated excellent agreement, 0.75–0.90 indicated good agreement, 0.75–0.50 indicated moderated agreement and <0.50 poor agreement [39]. All statistical analyses were performed using SPSS software (version 20.0). A *p*‐value of <0.05 was considered statistically significant in all tests.

## RESULTS

**Figure 4:**
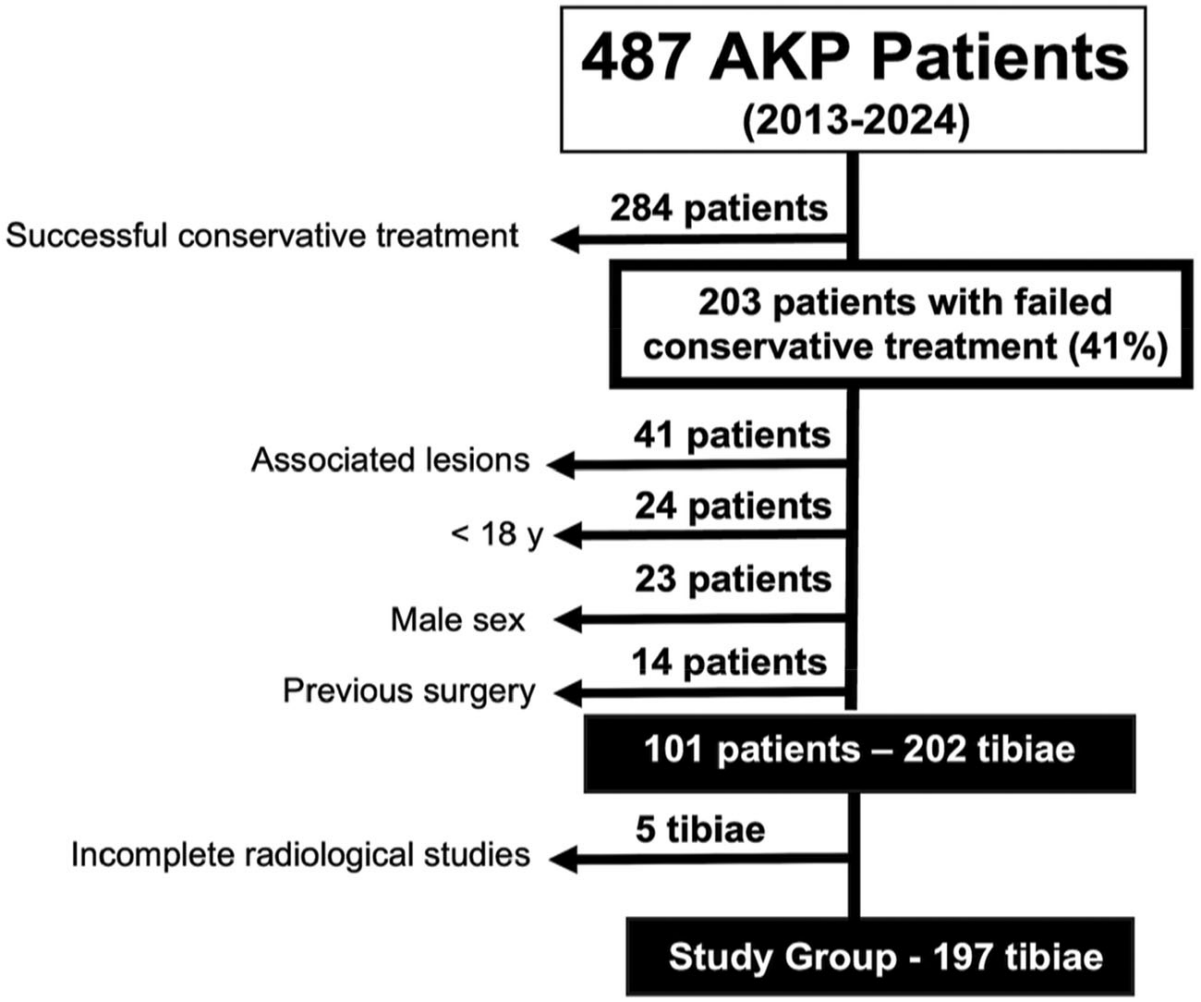
Flowchart of patient enrolment. Inclusion and exclusión criteria for the study group. AKP, anterior knee pain.

A total of 101 patients (197 tibiae) were included in this study. Flowchart of patient enrolment is shown in Figure 4. Details on patient demographics extracted from the hospital′s database are reported in Table 1. Detailed measured parameters are shown in Table 2. Normal ETT (≤30°) was observed in 24 tibiae, representing 12% of the total study group. Moderate ETT (31°−40°) was observed in 97 (49%), and severe ETT (>40°) in 76 (39%) (Table 2).

**Table 1:** Demographic and clinical characteristics of the patient cohort.

Patients (*n*)	101 (197 tibiae)
Age (years)	22.7 ± 5.9 (18–47)
Length of nonoperative management (months)	7.7 ± 1.7 (6–12)
BMI	20.5 ± 2.4 (18.2–33.8)

**Table 2:** Measurements of the study groups.

Grade of ETT	Number of tibiae	TTT	PTT	DTT	% DTT	TTL
Severe (>40°)	76	45.5° ± 4.1	29.4° ± 5.5	16.1° ± 5.8	35.2 ± 11.5	0.66 ± 0.04
Moderate (31°–40°)	97	35.7° ± 2.9	27.6° ± 3.8	8.0° ± 4.7	22.1 ± 12.1	0.65 ± 0.05
Normal (≤30°)	24	27.8° ± 3.8	22.6° ± 6.8	5.2° ± 3.7	18.2 ± 13.1	0.65 ± 0.03

*Note*: Values are expressed in mean ± SD. The percentage of DTT is the ratio of participation of tibial torsion distal to the TT on the TTT.

Abbreviations: DTT, tibial torsion distal to the TT; ETT, external tibial torsion; PTT, tibial torsion proximal to the TT; SD, standard deviation; TT, tibial tubercle; TTL, tibial tubercle lateralisation; TTT, total tibial torsion.

**Figure 5:**
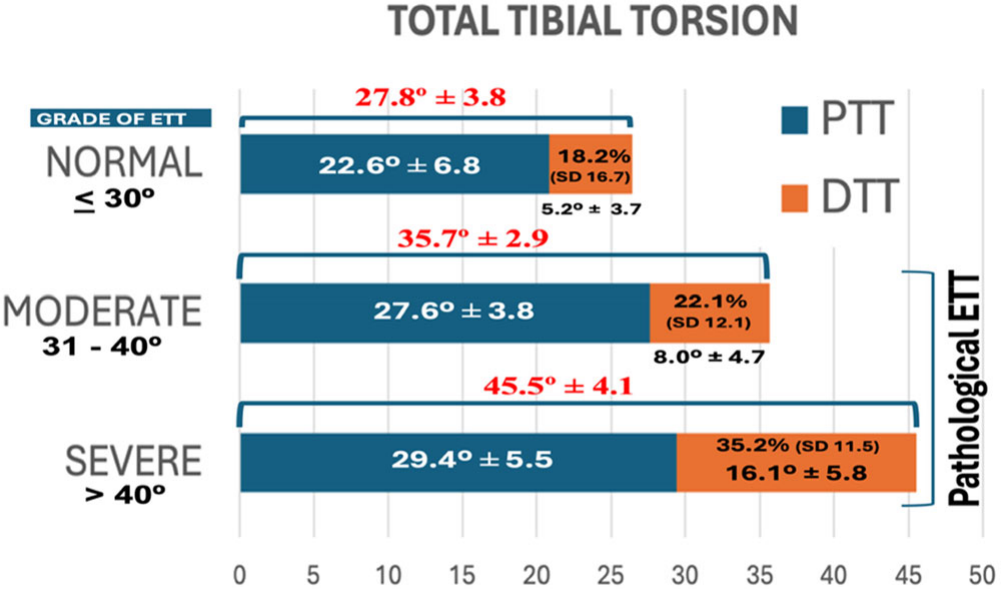
Distribution of the participation of proximal tibial torsion (PTT) and distal tibial torsion (DTT) in total tibial torsion (TTT) in the three study groups.

**Figure 6:**
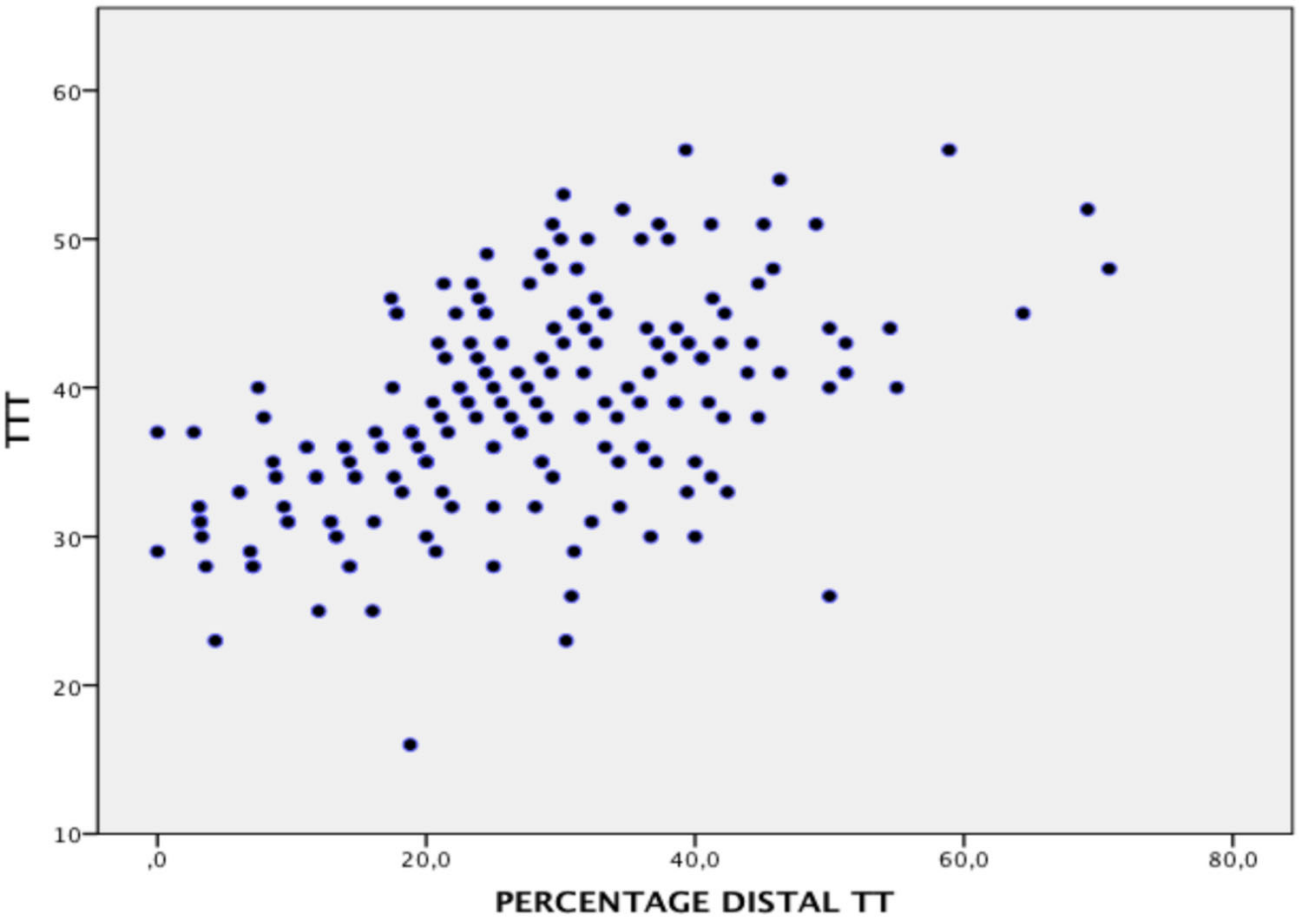
Scatter plot of the relationship between TTT and the participation of DTT expressed as a percentage of TTT. DTT, distal tibial torsion; TTT, total tibial torsion.

The mean tibial torsion angles were 27.8° (SD 3.8) in the control group, 35.7° (SD 2.9) in the moderate‐torsion group, and 45.5° (SD 4.1) in the high‐torsion group. Table 2 shows the data on the participation of the tibia proximal and distal to the TT in total tibial torsion. The increment of pathological ETT is the result of an increment in both supratuberositary and infratuberositary torsion (Figure 5). However, the percentage of contribution of infratuberositary torsion to the TTT was 18.2% (SD 16.7) in the control group, 22.1% (SD 12.1) in the moderate‐torsion group, and 35.2% (SD 11.5) in the high‐torsion group (*p* < 0.01 for the high‐torsion group) (Figure 5). A moderate statistically significant correlation was found between the degree of TTT and the percentage of contribution of DTT (*R* = 0.540, *p* < 0.001, *n* = 197). The linear regression analysis showed that ETT was a statistically significant predictor of the percentage of DTT (*p* < 0.001) (Figure 6). The increase in ETT explained 29% of the variability of the percentage of participation of DTT in total tibial torsion. For every 1‐unit increase in TTT, the percentage of DTT increased by 1.062 units (*B* = 1.062, Beta = 0.540). The constant (intercept) was −13.996, also statistically significant (*p* = 0.003). These results suggest a moderate positive association between ETT and the percentage of DTT in the TTT.

The control group showed a TTL ratio of 0.65 (SD 0.03), while the pathological group (severe and moderate) had a ratio of 0.65 (SD 0.05), with no statistically significant difference. A very mild correlation was found between ETT and TTL (*R* 0.166, *p* = 0.02). The linear regression model, with TTL value as the dependent variable and ETT as the independent variable, demonstrated poor predictive capacity, with an R‐squared value of 0.03. This indicates that ETT accounts for only 3% of the variance in TTL.

Inter‐rater reliability was good to excellent for all measurements. The ICC values were 0.854 (CI 95% 0.810–0.888), 0.784 (CI 95% 0.723–0.833) and 0.932 (CI 95% 0.911–0.948) for TTT, DTT and TTL, respectively.

## DISCUSSION

The most important finding of the present study is that in patients with high‐grade ETT, the segment of the tibia distal to the TT contributes significantly more to the rotational deformity compared to both the normal control group and patients with moderate ETT. Another relevant finding of this study is that the TT is not lateralized in female AKP patients with increased ETT.

Numerous studies have aimed to define the normal range of ETT in young, asymptomatic individuals [17, 19, 34, 36, 41]. Based on anthropometric data, cadaveric analyses, and imaging studies, a normal range for ETT in individuals of European origin is generally considered to be between 24° and 30° [36]. However, significant variability exists due to differences in the age of anatomical specimens, patient demographics, and variations across racial and age groups. Currently, there is no established consensus on the threshold values that delineate normal and pathological ETT. Therefore, it is not possible to define study groups based solely on such thresholds.

Our study employs cut‐off values associated with surgical indication for rotational tibial osteotomy to categorise patients. In most published surgical series, a threshold of 30° has been widely accepted for surgical intervention, as osteotomy in these cases has demonstrated improvements in both clinical and measurement outcomes [3, 15, 16, 25]. However, other authors advocate for surgical intervention only beyond 40°, which may also serve as another reasonable alternative threshold [13]. Using these thresholds, we categorised patients into three groups for the purposes of our study. Group 1: normal ETT (≤30°), Group 2: moderate ETT (31° to 40°) and Group 3: severe ETT (>40°). The fact that the cut‐off point for establishing our three study groups is based on values from which surgery is indicated according to the medical literature does not mean that we assign a surgical solution to a number. It is important to emphasise that in this cohort of patients surgery is indicated only in cases with severe and disabling AKP that is recalcitrant to conservative treatment. However, the debate about surgical indication is beyond the scope of this study. Our study is not a study of surgical indications. It is only an investigational anatomic imaging study.

Our results demonstrate that the increment in pathological ETT is attributed to increased torsion both proximal and distal to the TT. However, the percentage contribution of DTT to the TTT significantly increases with the severity of ETT. Specifically, the DTT contribution was 18.2% in the control group, 22.1% in the moderate‐torsion group, and 35.2% in the high‐torsion group (*p* < 0.01 for the high‐torsion group). This suggests that distal torsion plays a progressively larger role in severe cases of ETT. A moderate positive correlation (*R* = 0.540, *p* < 0.001, *n* = 197) between the degree of TTT and the percentage of DTT contribution further supports the increasing relevance of distal torsion in higher degrees of tibial torsion. From a clinical perspective, these findings suggest that in patients with severe ETT, the contribution of distal torsion becomes substantial, making it a critical factor in the deformity. This has direct implications for surgical planning. Performing a rotational osteotomy below the TT may address the distal component of the deformity more effectively.

In our study we have used the transverse ratio to measure TT lateralisation. Some authors have proposed the tibial tuberosity–trochlea groove (TT–TG) distance as the gold standard to determine the position of the TT [41]. However, the TT–TG distance depends on the tibiofemoral rotation [5, 18, 28, 35] and without accounting this, the measurement of the position of the TT on the tibia would be an invalid measurement. For instance, Tensho et al. [39] found that there was a correlation between the TT–TG distance and knee rotation, while Camathias et al. [5], have shown that rotation of the knee joint significantly altered the TT–TG value. Similarly, Pace et al. [28] found that the TT–TG distance is a multifactorial measurement, with factors such as external tibial rotation and trochlear dysplasia contributing more to elevated values than the lateral position of the TT itself. In this regard, another study showed that the average increase in TT–TG distance for each 1° of external tibial rotation was 0.55 mm (range: 0.50–0.62 mm) [35]. Further, Paiva et al. [29] have shown that increased TT–TG distance is due to medialization of the TG and not lateralisation of the TT. Alternative measures, such as the tibial tubercle–posterior cruciate ligament (TT–PCL) distance, have been proposed to evaluate TT lateralisation [32]. However, Dong et al. found that TT–PCL distance does not reliably reflect the true lateralisation of the TT [11]. To address these limitations, the present study employs a method detailed by Tensho et al. in 2015 to evaluate TT lateralisation more accurately [39]. These authors evaluate TT lateralisation relative to the maximum transverse diameter of the tibial plateau. TTL is expressed as a ratio of this transverse diameter and the distance between the midpoint of the TT and the medial border of the proximal tibial condyle. We have demonstrated that it is a method with an excellent reliability. For this reason, the conclusions we draw with this measurement are more reliable than those obtained using TT–TG distance.

The results of our study suggest that there is no significant difference in the TTL ratio between the control group and the pathological group (severe and moderate), as both groups exhibited comparable mean values of 0.65, with minimal variation. This finding implies that the degree of lateralisation of the TT may not be strongly influenced by the pathological condition under investigation. Additionally, while the correlation analysis revealed a very mild positive correlation between ETT and TTL (*R* = 0.166, *p* = 0.02), the strength of this relationship is negligible and of limited clinical relevance. The linear regression model further supports this conclusion, showing poor predictive capacity with an R‐squared value of 0.03. This indicates that ETT explains only 3% of the variance in TTL. These findings challenge the assumption that increased ETT is a primary determinant of TT lateralisation in this population [12]. In accordance with other clinical studies [2, 10, 21], our findings provide further evidence that increased ETT is not associated with a lateralized position of the TT. Moreover, the present study could not find a correlation between total or segmental tibial torsion and the lateralisation of the TT. Therefore, it makes little sense in our cohort of patients to make the osteotomy supratuberositary because we would be medializing a normopositioned TT.

### Limitations

The primary limitation of this study is the absence of a control group composed of individuals without patellofemoral disorders. Instead, the control group was derived from AKP patients who underwent torsional CT scans due to clinical suspicion of abnormal femoral and/or tibial torsion. For this study, tibiae with tibial torsion values considered normal (≤30°) were included as the control group. Consequently, the results cannot be generalised to healthy individuals without AKP. Furthermore, the study sample was limited to females, which restricts the applicability of the findings to male populations. Additionally, the results do not extend to individuals with pathological ETT who do not present with AKP. Therefore, the conclusions drawn from this study are applicable only to young females with AKP, and caution should be exercised when considering these findings in other populations. Future research is needed to evaluate whether the observed patterns differ in individuals with pathological ETT but without AKP, as well as in broader and more diverse populations, including males and healthy individuals.

## CONCLUSION

In females with AKP, the infratuberositary contribution to pathological ETT increases with the severity of the torsion. Moreover, the degree of ETT does not affect TT lateralisation.

## AUTHOR CONTRIBUTIONS

**Vicente Sanchis‐Alfonso**: Project coordinator; designing the study; writing the manuscript; evaluating the radiographs; collecting and analysing the data. **Cristina Ramírez‐Fuentes**: Writing the manuscript; evaluating the radiographs; collecting data. **Jose Yañez‐Rodríguez**: Evaluating the radiographs. **Laura Parra‐Calabuig** and **Marcos López‐Vega**: Collecting data. **Julio Domenech‐Fernandez**: Writing the manuscript and analysing the data.

## CONFLICT OF INTEREST STATEMENT

The authors declare no conflicts of interest.

## ETHICS STATEMENT

The study was approved by the clinical research ethics committee at our institution (CEIm Hospital Arnau de Vilanova, Valencia, Spain # PI 21_2024) and conducted in accordance with the ethical standards laid down in the 1964 Declaration of Helsinki and its later amendments. Informed consent was obtained from all individual participants included in the study.

## Data Availability

The data that support the findings of this study are available from the corresponding author upon reasonable request.
